# Arachidonic Acid and Docosahexaenoic Acid Suppress Osteoclast Formation and Activity in Human CD14+ Monocytes, *In vitro*

**DOI:** 10.1371/journal.pone.0125145

**Published:** 2015-04-13

**Authors:** Abe E. Kasonga, Vishwa Deepak, Marlena C. Kruger, Magdalena Coetzee

**Affiliations:** 1 Department of Physiology, Faculty of Health Sciences, University of Pretoria, Pretoria, South Africa; 2 School of Food and Nutrition, Massey Institute of Food Science and Technology, Massey University, Palmerston North, New Zealand; 3 Department of Human Nutrition and Associate of the Institute for Food, Nutrition and Well-being, University of Pretoria, Pretoria, South Africa; Max Delbrueck Center for Molecular Medicine, GERMANY

## Abstract

An unbalanced diet can have adverse effects on health. Long chain polyunsaturated fatty acids (LCPUFAs) have been the focus of research owing to their necessity of inclusion in a healthy diet. However, the effects of LCPUFAs on human osteoclast formation and function have not been explored before. A human CD14+ monocyte differentiation model was used to elucidate the effects of an ω-3 LCPUFA, docosahexaenoic acid (DHA), and an ω-6 LCPUFA, arachidonic acid (AA), on osteoclast formation and activity. CD14+ monocytes were isolated from peripheral blood of healthy donors and stimulated with macrophage colony stimulating factor and receptor activator of nuclear factor kappa-B ligand to generate osteoclasts. Data from this study revealed that both the LCPUFAs decreased osteoclast formation potential of CD14+ monocytes in a dose-dependent manner when treated at an early stage of differentiation. Moreover, when exposed at a late stage of osteoclast differentiation AA and DHA impaired the bone resorptive potential of mature osteoclasts without affecting osteoclast numbers. AA and DHA abrogated vitronectin receptor expression in differentiating as well as mature osteoclasts. In contrast, the degree of inhibition for calcitonin receptor expression varied between the LCPUFAs with only AA causing inhibition during osteoclast differentiation. Furthermore, AA and DHA down regulated the expression of key osteoclast-specific genes in differentiating as well as mature osteoclasts. This study demonstrates for the first time that LCPUFAs can modulate osteoclast formation and function in a human primary osteoclast cell line.

## Introduction

Bone is a dynamic tissue that is constantly remodelled by osteoclasts and osteoblasts. In healthy adults, bone resorption by osteoclasts is usually followed by bone formation by osteoblasts in a balanced manner [1]. An imbalance in the process occurs during bone-related diseases such as osteoporosis, hypocalcaemia or osteopetrosis [1, 2]. Therefore, intercellular communication between the osteoblasts and osteoclasts is crucial in maintaining the structure of the bone tissue. Osteoclasts, the sole bone resorbing cell in the body, are multinuclear, terminally differentiated cells that are derived from haematopoietic precursors of the monocyte/macrophage lineage [3, 4, 5, 6]. Receptor activator of nuclear factor kappa-B ligand (RANKL) and macrophage colony stimulating factor (M-CSF), which are both produced by osteoblasts, induce osteoclast precursors to differentiate and fuse into resorbing osteoclasts [7]. M-CSF is responsible for the proliferation, differentiation and survival of osteoclast precursors while RANKL stimulates osteoclastogenesis and prevents osteoclast apoptosis [4, 5, 8, 9]. RANKL signalling activates a cascade of signalling events that leads to activation and expression of certain transcription factors and markers indispensable for osteoclast formation. The transcription factors include c-Fos and NFATc1, whereas the osteoclast-specific markers include tartrate resistant acid phosphatase (TRAP), cathepsin K (CTSK), matrix metalloproteinase 9 (MMP-9) and dendritic cell-specific transmembrane protein (DC-STAMP) [10].

At sites of bone contact, the osteoclast forms F-actin rings and a sealing zone between the bone and the osteoclast which is mediated by integrin α_ν_β_3_, also known as vitronectin receptor (VNR) [1, 11]. Inside the sealing zone, osteoclasts form a specialized cell membrane known as the ruffled border that facilitates the resorptive function of osteoclasts [1, 11]. Once osteoclasts attach to bone, carbonic anhydrase (CA) acidifies the resorption micro-environment to dissolve the mineral phase of bone [1, 11]. The lysosomal enzymes, CTSK and MMP-9, are released to degrade the organic matrix [2, 3, 11]. The degradation products are then endocytosed by the osteoclasts and released into the extracellular fluid [1, 7].

Long chain polyunsaturated fatty acids (LCPUFAs) are fatty acids with a minimum of 18 carbons and 2 double bonds that can be categorized into two principal families, namely: ω-3 LCPUFAs and ω-6 LCPUFAs [4]. The anti-inflammatory ω-3 LCPUFAs are derived from α-linolenic acid (ALA) while the pro-inflammatory ω-6 LCPUFAs are derived from linoleic acid (LA) [4, 8, 12, 13]. The human body is unable to synthesize fatty acids containing double bonds after carbon 9 (from the carboxyl end), hence both ALA and LA are categorised as essential fatty acids and must be supplied in the diet [4, 8]. A series of shared enzymes can metabolize LA and ALA into several different metabolites. The most common metabolites of LA and ALA in the human body are arachidonic acid (AA) and docosahexaenoic acid (DHA) respectively [14].

A LCPUFA-enriched diet has been shown to decrease the risk of hip fractures in older adults [15]. Beneficial effects of ω-3 LCPUFAs on animal [8, 12, 16, 17] and human [18, 19, 20] bone have been reported. ω-6 LCPUFAs (particularly AA) have also been shown to be favourable for healthy bone maintenance [21]. Some studies have shown less promising effects of LCPUFAs on bone health [22, 23]. The interpretation of these results is challenging due to environmental, genetic and even dietary factors, such as the differences in the source of certain fatty acids, which are unique to specific regions [24].

*In vitro* studies on animal cell lines have shown that DHA can inhibit murine osteoclast formation [25, 26] as well as osteoclast-specific gene expression such as TRAP, MMP-9, CTSK [27] and calcitonin receptor (CTR) [28]. AA has been shown to have osteoclast inducing [26] as well as osteoclast inhibitory effects on murine cells [27]. Employment of dissimilar research conditions and RANKL concentrations can contribute to the contradictory results. *In vitro* studies on animal cell lines have allowed better understanding of the possible positive or negative effects exerted by LCPUFAs on bone. However, studies analysing the effects of LCPUFAs on human osteoclast progenitors and their osteoclastogenic potential are still lacking. To the best of our knowledge this study for the first time explores the effects of LCPUFAs on human osteoclast formation from CD14+ monocytes. Additionally, this study aims to provide more precise information on the LCPUFA-responses that may occur in bone cells, *in vitro*.

## Materials and Methods

### Reagents and materials

α-MEM was obtained from GIBCO (Invitrogen Corp, Australia) and heat inactivated fetal calf serum (FCS) was purchased from Amersham (Little Chalfont, UK). Antibiotic-antimycotic solution containing 100 U ml^-1^ penicillin, 100 μg ml^-1^ streptomycin and 0.25 μg ml^-1^ fungizone was supplied by Highveld Biological (Johannesburg, South Africa). DHA, AA, Histopaque, TRI Reagent and all other chemicals of research grade were obtained from Sigma-Aldrich Inc. (St Louis, MO, USA). RANKL and M-CSF were acquired from Insight Biotechnology (Middlesex, UK) and R&D Systems (Minneapolis, MN, USA) respectively. RANKL and M-CSF were prepared in α-MEM, aliquoted and stored at -20°C and -70°C respectively until further use. All components for the magnetic separation of CD14+ monocytes were supplied by Miltenyi Biotec (San Diego, CA, USA). Alamar blue reagent, cell extraction buffer, 4–12% NuPAGE Novex Bis-Tris precasted polyacrylamide gels, iBlot Gel Transfer Device, iBlot Western Detection Chromogenic Kit, Alexa Fluor 568-Phalloidin and Alexa Fluor 488-goat anti-mouse antibody (#A-11001) were supplied by Life Technologies (Carlsbad, CA, USA). The bicinchoninic acid (BCA) protein assay kit was purchased from Thermo Scientific (Rockford, IL, USA). M-MuLV reverse transcriptase was purchased from New England Biolabs (Hitchin, UK). KAPA SYBR FAST qPCR Master Mix was purchased from Kapa Biosystems (Cape Town, South Africa). Rabbit polyclonal antibodies against GAPDH (#37168), MMP-9 (#38898), CTSK (#19027) and TRAP (#96372) were procured from Abcam (Cambridge, MA, USA). Mouse monoclonal antibodies against VNR (#MAB3050) and CTR (#MAB4614) were purchased from R&D Systems (Minneapolis, MN, USA).

### Fatty acid preparation

AA and DHA were prepared in ethanol at a stock concentration of 100 mM, aliquoted and stored at -70°C until required. Stock solutions were freshly diluted to working concentrations in complete culture medium just before the experiments. The highest concentration of ethanol did not exceed 0.08% in the experiments. Ethanol at 0.08% did not show any harmful effects on the cells tested in our laboratory and this concentration was used for the vehicle control.

### Ethics statement

All the procedures and experimental protocols used in this study were approved by the Human Research Ethics Committee of the Faculty of Health Sciences, University of Pretoria (Protocol approval number: S154/2012). Eligible participants were asked to provide an additional written informed consent for enrolment.

### Isolation of CD14+ monocytes and cell culture

Human CD14+ monocytes were isolated as previously described [29]. Briefly, 40–60 ml of peripheral blood was collected from healthy male donors (participants, aged 18–35) into heparinized tubes. Peripheral blood mononuclear cells were separated from peripheral blood by centrifugation on a Histopaque gradient and CD14+ monocytes were isolated using CD14+ magnetic beads [29].

CD14+ monocytes were seeded at a density of 1.3 x 10^5^ cells cm^-2^ and cultured in α-MEM containing 10% FCS and antibiotic-antimycotic solution. The media was supplemented with 25 ng ml^-1^ M-CSF and 30 ng ml^-1^ RANKL to stimulate osteoclast differentiation. Cells were maintained at 37°C in a humidified atmosphere of 93% air and 7% CO_2_ for 3 weeks as described by Agrawal et al [29]. For the experiments involving analysis of the effects of LCPUFAs on differentiating osteoclasts, cells were either maintained in medium containing differentiation factors and various concentrations of LCPUFAs or the vehicle (0.08% ethanol) from day 3. For the experiments involving analysis of the effects of LCPUFAs on mature osteoclast activity, cells were exposed to differentiation factors in the presence of the vehicle (0.08% ethanol) or LCPUFAs from the onset of resorption (typically day 14). Medium and all factors were replaced every 2–3 days.

### Alamar blue assay

CD14+ monocytes were seeded on glass cover-slips placed in 96-well plates. Cells were exposed to M-CSF in combination with increasing concentrations of AA or DHA (20 μM, 40 μM, 60 μM and 80 μM) or the vehicle (0.08% ethanol) for 48 h. Alamar blue assay was conducted according to the manufacturer’s instructions. Optical absorbance was measured at 570 nm with 600 nm as the reference using an Epoch Micro-plate Spectrophotometer (BioTek, Winooski, VT, USA).

### Quantification of tartrate-resistant acid phosphatase (TRAP) in conditioned media

CD14+ monocytes were seeded on glass cover-slips placed in 96-well plates. Cells were exposed to differentiation factors in combination with increasing concentrations of AA or DHA (20 μM, 40 μM, 60 μM and 80 μM) or the vehicle (0.08% ethanol) as mentioned before. At the end of culture, TRAP activity in the media was measured as previously described [30]. In brief, the conditioned medium was collected from each well and incubated at 37°C in 6 mM para-nitrophenylphosphate, in the presence of 25 mM disodium tartrate at pH 5.5 for an hour. The reaction was stopped with the addition of 0.3 M sodium hydroxide. TRAP activity was quantified by measuring optical absorbance at 405 nm with 650 nm as the reference using an Epoch Micro-plate Spectrophotometer (BioTek, Winooski, VT, USA).

### Quantification of tartrate-resistant acid phosphatase positive (TRAP+) stained osteoclasts

CD14+ monocytes were seeded on glass cover-slips in 96-well plates and exposed to differentiation factors in combination with increasing concentrations of LCPUFAs (20 μM, 40 μM, 60 μM and 80 μM) or the vehicle (0.08% ethanol) as mentioned before. At the end of the culture period, osteoclasts were fixed with 10% formalin and stained for the presence of TRAP using a modified TRAP staining protocol [29]. Briefly, the fixative was removed and warm acetate-tartrate buffer (0.1 M sodium tartrate in 0.2 M acetate buffer, pH 5.2) was added to the cells for 5 min at 37°C. The buffer was removed and the cells were incubated at 37°C in 20 mg ml^-1^ naphthol AS-BI phosphate in acetate-tartrate buffer for 30 min. The solution was removed and acetate-tartrate buffer hexazotised pararosaniline solution was added. After 15 min incubation at 37°C, the cells were counter-stained with haematoxylin for 40 s, rinsed, dried and visualised by light microscopy. Osteoclasts appeared as large multinucleated cells staining red [7]. The effect of the LCPUFAs on osteoclast formation was determined by counting the number of TRAP-positive stained cells with three or more nuclei per unit area [28] and comparing that with the number of TRAP-positive cells counted in the vehicle control. Photomicrographs were taken with a Zeiss Axiocam MRc5 camera attached to a Zeiss Axiovert 40 CFL microscope (Carl Zeiss AG, Oberkochen, Germany).

### Quantification of bone resorption pit formation on dentine discs

CD14+ monocytes were seeded on dentine discs in 96-well plates and exposed to differentiation factors in combination with increasing concentrations of LCPUFAs (20 μM, 40 μM, 60 μM and 80 μM) or vehicle control as mentioned before. At the end of the culture period, the osteoclasts were fixed with 10% formalin and TRAP stained as previously mentioned. Haematoxylin was used for counterstaining as well as for staining the resorption pits on the dentine discs. Dentine discs were imaged using a Zeiss Axiocam MRc5 camera attached to a Zeiss SteREO Discovery.V8 microscope (Carl Zeiss AG, Oberkochen, Germany). Resorption was determined by point counting [31]. The method was slightly modified with the use of Image J software [32] to create a grid. Resorption pits crossing the intersecting points on the grid were counted.

### Cell morphology—Polarization-optical differential interference contrast (PlasDIC) microscopy

CD14+ monocytes were seeded on glass cover-slips in 96-well plates and exposed to LCPUFAs (40 μM) or vehicle control in the presence of differentiation factors. PlasDIC images were obtained with a Zeiss Axiocam MRc5 camera attached to a Zeiss Axiovert 40 CFL microscope (Carl Zeiss AG, Oberkochen, Germany).

### Immunofluorescence microscopy

Visualization of actin ring, VNR and CTR expression was analysed by immunofluorescence microscopy. CD14+ monocytes were seeded on glass cover-slips in 24-well plates and exposed to LCPUFAs (40 μM) or vehicle control in the presence of differentiation factors. At the end of culture cells were stained for actin, VNR and CTR as described previously [33]. Briefly, cells were washed twice with PBS and fixed with 3.7% (v/v) formaldehyde in PBS for 15 min. Cells were permeabilised for 5 min with 0.1% Triton X-100 and stained for actin with 5 U/ml Alexa Fluor 568-Phalloidin and 35 μg/ml Hoechst for nucleus. For VNR or CTR staining, 50 μg/ml mouse anti-VNR or anti-CTR antibodies were employed followed by staining with 2 μg/ml Alexa Fluor 488-goat anti-mouse secondary antibodies. Images were acquired by confocal laser scanning microscopy using a Zeiss Axiocam MRc5 camera attached to a Zeiss Axiovert 40 CFL microscope (Carl Zeiss AG, Oberkochen, Germany) using the appropriate filter sets: Hoechst (Excitation: 352 nm, Emission: 455 nm); Alexa Fluor 488 (Excitation: 490 nm, Emission: 525 nm) Alexa Fluor 568-Phalloidin (Excitation: 578 nm, Emission: 600 nm).

### Immunoblotting

CTSK, TRAP and MMP-9 protein expression was evaluated by immunoblotting [27]. CD14+ monocytes were seeded on glass cover-slips in 24-well plates and exposed to LCPUFAs (40 μM) or vehicle control in the presence of differentiation factors as mentioned before. At the end of culture cells were lysed with cell extraction buffer. Resultant lysates were centrifuged for 30 min at 13 000 g and 4°C for the removal of cell debris. Supernatants were collected and protein concentration was determined using a BCA protein concentration determination kit. Equal amount of proteins were loaded onto each lane and resolved on a 4–12% NuPAGE Novex Bis-Tris precasted polyacrylamide gel, and subsequently electrotransferred onto a nitrocellulose membrane using an iBlot Gel Transfer Device at 21 volts for 1 min, 23 volts for 4 min and 25 volts for 2 min. The membranes were blocked for non-specific binding by incubation at room temperature in 5% skim milk powder for 1 h, followed by incubation with rabbit polyclonal antibodies against GAPDH, MMP-9, CTSK, TRAP (1:1000) overnight at 4°C. Membranes were then incubated with agitation in alkaline-phosphatase conjugated secondary antibodies (1:1000) for 1 h. iBlot Western Detection Chromogenic Kit was used to develop the blots and digital images of the blots were obtained by using a flatbed scanner (Ricoh Aficio, Johannesburg, South Africa).

### Gene expression analysis of osteoclast-specific markers

CD14+ monocytes were seeded on glass cover-slips in 24-well plates and exposed to LCPUFAs (40 μM) or vehicle control in the presence of differentiation factors as mentioned before. Total RNA was extracted using TRI Reagent and reverse transcribed to cDNA using M-MuLV reverse transcriptase. KAPA SYBR FAST qPCR Master Mix was used for the quantitative real-time PCR (qRT-PCR) assay. The 2^-ΔΔCT^ method was used to analyse relative gene expression levels and the results were normalized to the housekeeping gene (GAPDH) [34]. All the primers used in the study were synthesized by Inqaba Biotec (Pretoria, South Africa) and are illustrated in Table 1.

**Table 1:** Primers used in this study.

Gene	Forward Primer	Reverse Primer
c-Fos	5’ CCCATCGCAGACCAGAGC 3’	5’ ATCTTGCAGGCAGGTCGGT 3’
NFATc1	5’ GTGGAGAAGCAGAGCAC 3’	5’ ACGCTGGTACTGGCTTC 3’
CA2	5’ GAGTTTGATGACTCTCAGGACAA 3’	5’ CATATTTGGTGTTCCAGTGAACCA 3’
DC-STAMP	5’ ATGACTTGCAACCTAAGGGCAAAG 3’	5’ GTCTGGTTCCAAGAAACAAGGTCAT 3’
RANK	5’ TCTGCTTCTCTTCGCGTCTG 3’	5’ CGTAGGGACCACCTCCTACA 3’
GAPDH	5’ GATGACATCAAGAAGGTGGTGAAGC 3’	5’ATACCAGGAAATGAGCTTGACAAAG 3’

### Statistics

Data are representative of three independent experiments conducted in triplicate and are expressed as mean ±SD (standard deviation) unless otherwise stated. Data were analysed using one or two way analysis of variance (ANOVA) as appropriate followed by Bonferroni post hoc test using GraphPad Prism software (GraphPad software Inc, California, USA). Testing was done at the 0.05 level of significance.

## Results

### Effects of arachidonic acid and docosahexaenoic acid on TRAP activity

**Fig 1:**
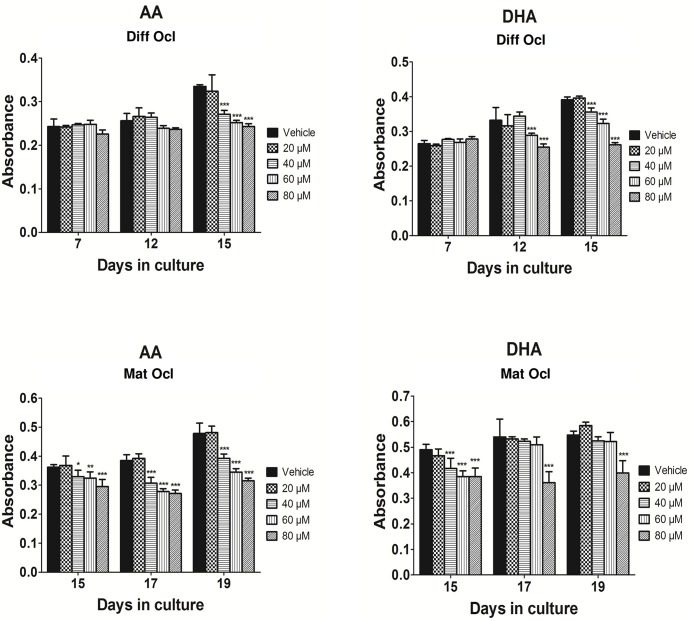
Effects of AA and DHA on TRAP activity. Osteoclast differentiation was stimulated in CD14+ monocytes by the addition of 25 ng ml^-1^ M-CSF and 30 ng ml^-1^ RANKL as described in Materials and Methods. Vehicle (0.08% ethanol) or LCPUFAs were added from day 3 in differentiating osteoclasts and from the onset of resorption (day 12–14) in mature osteoclasts. At the time points indicated, the conditioned medium was removed and analysed for TRAP activity using para-nitrophenylphosphate as a substrate. TRAP activity was measured in: A. differentiating osteoclasts exposed to AA, B. differentiating osteoclasts exposed to DHA, C. mature osteoclasts exposed to AA and D. mature osteoclasts exposed to DHA. The results are representative of three independent experiments conducted in triplicate. Diff Ocl—differentiating osteoclasts. Mat Ocl—mature osteoclasts. *P<0.05; **P<0.01; ***P<0.001 vs. vehicle.

Differentiating and mature osteoclasts show high levels of TRAP activity which is commonly used as a marker for osteoclastogenesis [35]. We sought to determine whether AA and DHA could affect TRAP activity in a time or dose-dependent manner in differentiating and mature osteoclasts. AA and DHA were tested at concentrations of 20 μM, 40 μM, 60 μM and 80 μM respectively. At these concentrations, the LCPUFAs did not exert any cytotoxic effects on the human CD14+ monocytes (S1 Fig). In differentiating osteoclasts, AA significantly decreased TRAP activity at concentrations of 40 μM and above from day 15 (Fig 1A). On the other hand, DHA at concentrations of 60 μM and above suppressed TRAP activity as early as day 12 in differentiating osteoclasts (Fig 1B). When mature osteoclasts were exposed to AA at day 12 and analysed for enzymatic activity at day 15, the LCPUFA at 40 μM and above potently exacerbated TRAP activity (Fig 1C). This trend continued to follow even at the 17th and 19th day of osteoclast differentiation. Similarly, DHA treated mature osteoclasts (exposed to DHA from day 12) at day 15 experienced a decline in TRAP activity at concentrations of 40 μM and above (Fig 1D). However, TRAP activity in DHA treated mature osteoclasts at day 17 and 19 did not show a significant decrease to the LCPUFA at concentrations lower than 80 μM (Fig 1D).

### Effects of arachidonic acid and docosahexaenoic acid on the osteoclast formation

**Fig 2:**
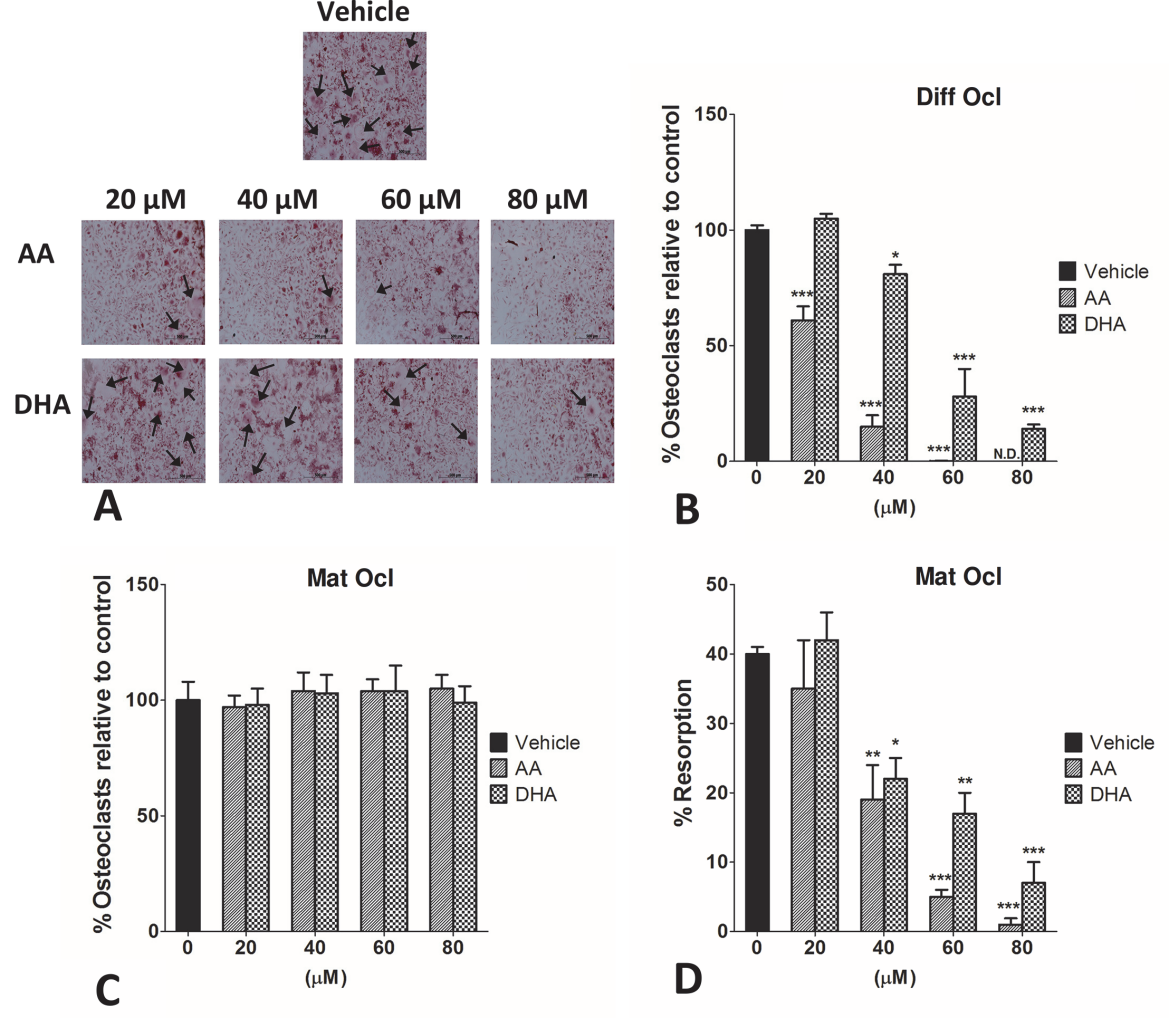
Effects of AA and DHA on osteoclast formation and resorption pit formation. CD14+ monocytes were seeded in 96-well plates and osteoclast differentiation was stimulated by the addition of 25 ng ml^-1^ M-CSF and 30 ng ml^-1^ RANKL as described in Materials and Methods. Vehicle (0.08% ethanol) or LCPUFAs were added from day 3 for in differentiating osteoclasts and from the onset of resorption (day 12–14) in mature osteoclasts. Experiments were terminated after 3 weeks. Osteoclasts were TRAP stained and counted under a light microscope. Haematoxylin was used to visualize the nuclei as well as the resorption pits on dentine discs. TRAP+ cells with 3 or more nuclei were counted. A. Photomicrographs of the effects of AA and DHA on osteoclast formation in differentiating osteoclasts seeded on glass coverslips. Large multinucleated osteoclasts are indicated with black arrows. Scale bar = 500 μm. B. Effects of AA and DHA on osteoclast formation in differentiating osteoclasts. C. Effects of AA and DHA on mature osteoclast numbers. D. Effects of AA and DHA on resorption pit formation on dentine discs in mature osteoclasts. The results are representative of three independent experiments conducted in triplicate. Diff Ocl—differentiating osteoclasts. Mat Ocl—mature osteoclasts. N.D.—No osteoclasts detected. *P<0.05; **P<0.01; ***P<0.001 vs. vehicle.

To determine whether AA and DHA could have a time or dose-dependent effect on osteoclastogenesis, TRAP+ osteoclasts were quantified. Differentiating or mature osteoclasts were exposed to both the LCPUFAs at increasing concentrations as mentioned before. AA and DHA significantly reduced RANKL-induced osteoclast formation in differentiating osteoclasts in a dose-dependent manner, as exposure to both LCPUFAs yielded fewer large multinucleated osteoclasts (Fig 2A). AA decreased osteoclast formation with a much higher potency than DHA. AA showed inhibitory effects at a concentration as low as 20 μM with complete inhibition of osteoclast formation at 80 μM. DHA on the other hand mitigated osteoclastogenesis at concentrations of 40 μM and above (Fig 2B). Interestingly, both the fatty acids failed to have an effect on TRAP+ mature osteoclast numbers when exposed to all the tested concentrations (20–80 μM) (Fig 2C).

As 40 μM was the lowest concentration at which both the LCPUFAs showed an inhibitory effect on osteoclastogenesis, this concentration was used for downstream experiments.

### Effects of arachidonic acid and docosahexaenoic acid on bone resorption

The unique feature of osteoclasts is to effectively resorb bone and degrade bone matrix. We next examined whether AA and DHA could affect bone resorption. Since, both the LCPUFAs blunted early osteoclastogenesis, resorption pit assays were not conducted on differentiating osteoclasts. Mature osteoclasts grown on dentine discs were exposed to AA or DHA at concentrations of 20–80 μM respectively. Intriguingly, both the LCPUFAs significantly (*p*<0.001) decreased resorption pit formation on dentine discs at concentrations of 40 μM and above (Fig 2D).

### Effects of arachidonic acid and docosahexaenoic acid on cell morphology

**Fig 3:**
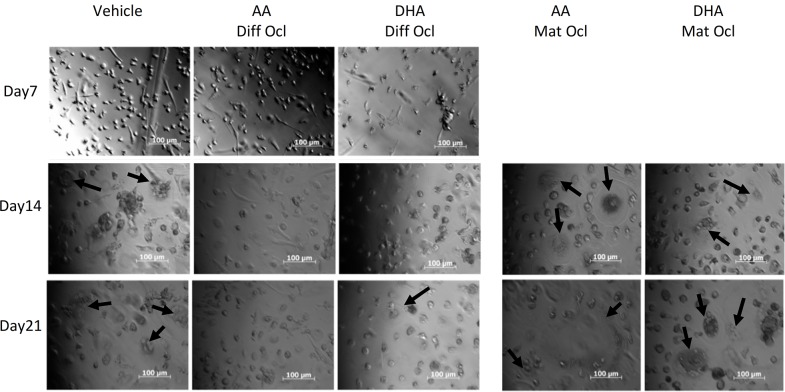
Effects of AA and DHA on osteoclast cell morphology. Osteoclast differentiation was stimulated in CD14+ monocytes by the addition of 25 ng ml^-1^ M-CSF and 30 ng ml^-1^ RANKL as described in Materials and Methods. Vehicle (0.08% ethanol) or LCPUFAs (40 μM) were added from day 3 in differentiating osteoclasts and from the onset of resorption (day 12–14) in mature osteoclasts. Osteoclasts were visualized under a microscope and PlasDIC images were taken throughout the culture period. Large multinucleated osteoclasts are indicated with black arrows. The results are representative of three independent experiments conducted in triplicate. Scale bar = 100 μm. Diff Ocl—differentiating osteoclasts. Mat Ocl—mature osteoclasts.

PlasDIC is an improved differential interference contrast method which provides high-quality imaging of cells grown in plastic cell culture containers. PlasDIC was used to observe morphological characteristics of CD14+ monocytes and osteoclasts derived from these monocytes during exposure to the LCPUFAs. Photomicrographs were taken from day 7 of culture. In differentiating osteoclasts exposed to either LCPUFA, no large multinucleated cells were seen on day 14. Fewer and smaller multinucleated cells could be seen in LCPUFA-exposed differentiating osteoclasts compared to the control on day 21. Mature osteoclasts were exposed to LCPUFAs from day 14, however they still formed large multinucleated cells and no morphological differences were seen between these cells and those of the control at day 21 (Fig 3).

### Effects of arachidonic acid and docosahexaenoic acid on actin ring formation and VNR and CTR expression

**Fig 4:**
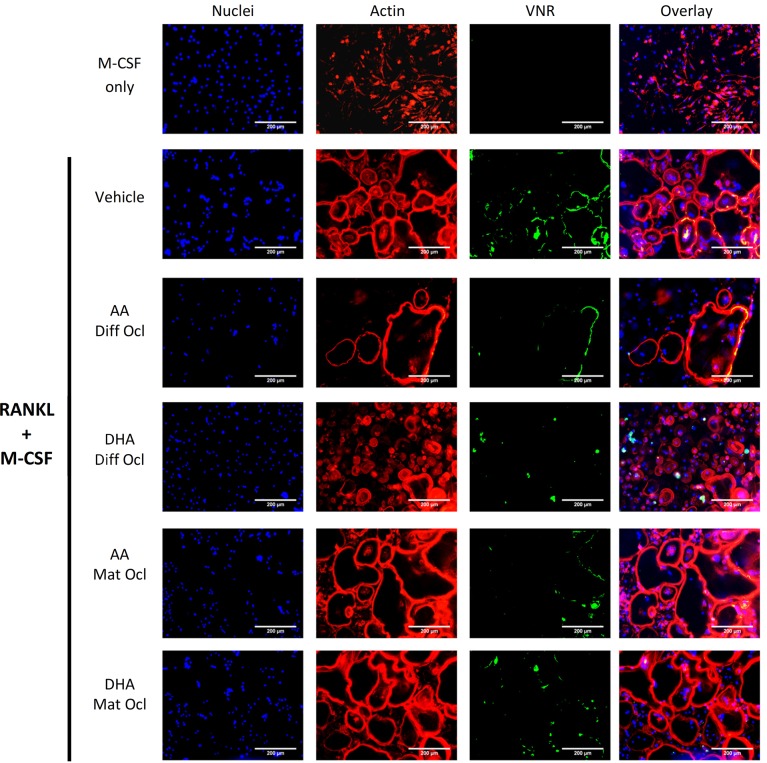
Effects of AA and DHA on actin ring formation and VNR expression. Osteoclast differentiation was stimulated in CD14+ monocytes by the addition of 25 ng ml^-1^ M-CSF and 30 ng ml^-1^ RANKL as described in Materials and Methods. Vehicle (0.08% ethanol) or LCPUFAs (40 μorption (day 12–14) in mature osteoclasts. After 3 weeks of culture, cells were fixed and stained with Hoechst (blue) for nuclei, phalloidin (red) for actin rings, and anti-VNR antibody (green). The results are representative of three independent experiments conducted in triplicate. Scale bar = 200 μm. Diff Ocl—differentiating osteoclasts. Mat Ocl—mature osteoclasts.

**Fig 5:**
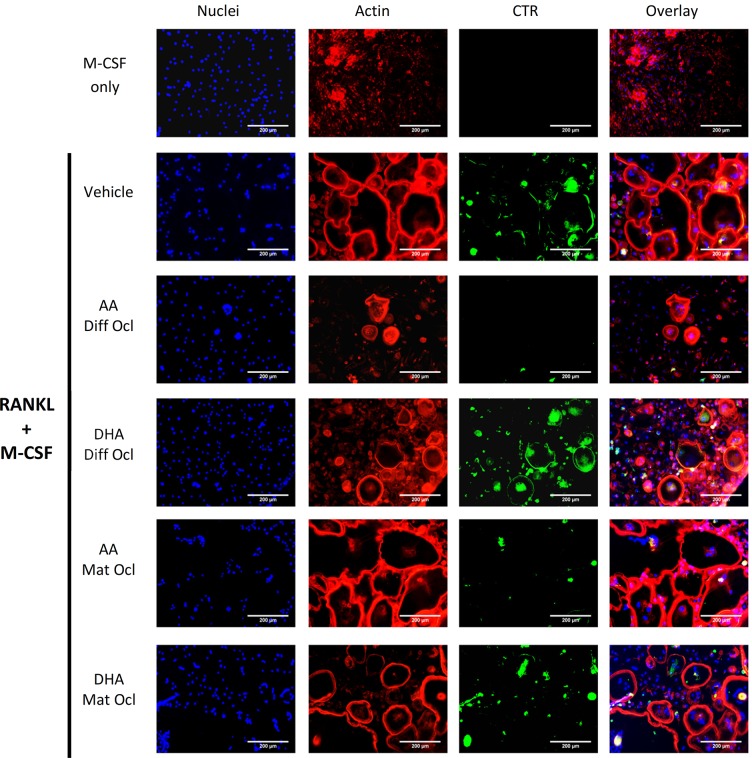
Effects of AA and DHA on actin ring formation and CTR expression. Osteoclast differentiation was stimulated in CD14+ monocytes by the addition of 25 ng ml^-1^ M-CSF and 30 ng ml^-1^ RANKL as described in Materials and Methods. Vehicle (0.08% ethanol) or LCPUFAs (40 μM) were added from day 3 in differentiating osteoclasts and from the onset of resorption (day 12–14) in mature osteoclasts. After 3 weeks of culture, cells were fixed and stained with Hoechst (blue) for nuclei, phalloidin (red) for actin rings, and anti-CTR antibody (green). The results are representative of three independent experiments conducted in triplicate. Scale bar = 200 μm. Diff Ocl—differentiating osteoclasts. Mat Ocl—mature osteoclasts.

VNR and CTR are cell surface receptors that are highly expressed in mature osteoclasts [3]. After 3 weeks of culture, cells were stained for actin, VNR and CTR. Although fewer and smaller osteoclasts were seen in the differentiating osteoclasts exposed to either LCPUFA (40 μM), when compared to the control, neither LCPUFA showed any effect on the formation of the actin rings (Figs 4 and 5). AA inhibited both VNR (Fig 4) and CTR (Fig 5) expression in differentiating and mature osteoclasts. DHA slightly inhibited VNR expression (Fig 4) in differentiating and mature osteoclasts, but showed no effect on CTR expression (Fig 5). In mature osteoclasts, the actin rings of the cells exposed to the LCPUFAs greatly resembled those in the control.

### Effects of arachidonic acid and docosahexaenoic acid on osteoclast specific marker expression

**Fig 6:**
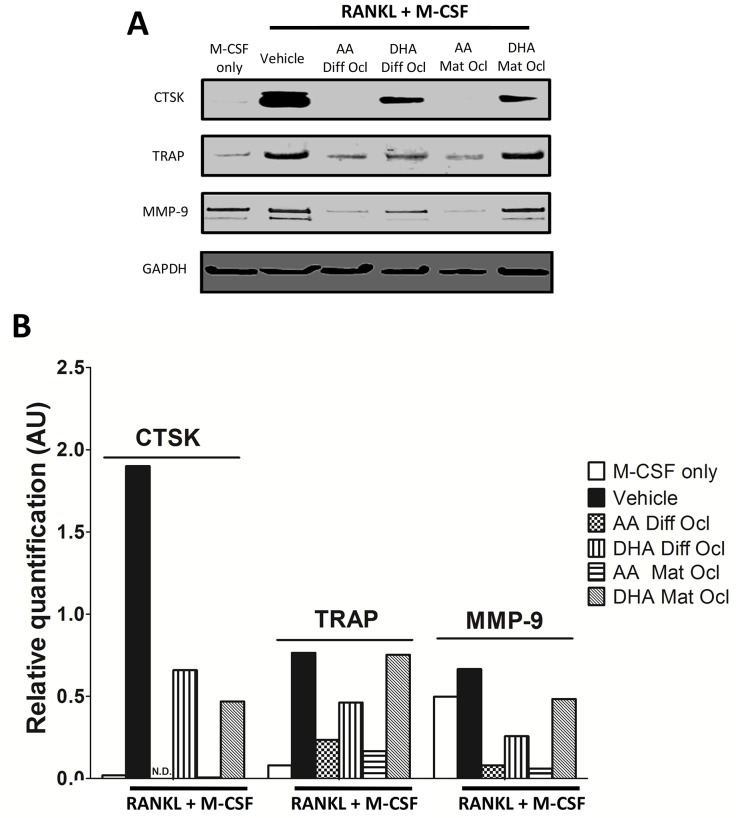
Effects of AA and DHA on protein expression. Osteoclast differentiation was stimulated in CD14+ monocytes by the addition of 25 ng ml^-1^ M-CSF and 30 ng ml^-1^ RANKL as described in Materials and Methods. Vehicle (0.08% ethanol) or LCPUFAs (40 μM) were added from day 3 in differentiating osteoclasts and from the onset of resorption (day 12–14) in mature osteoclasts. Experiments were terminated after 3 weeks. A. Protein expression for CTSK, TRAP and MMP-9 was analysed by western blot. B. Relative densities were determined using ImageJ software. The results are representative of two independent experiments conducted in triplicate. Diff Ocl—differentiating osteoclasts. Mat Ocl—mature osteoclasts. N.D.—Not detected.

We further analysed the effects of the tested LCPUFAs on osteoclast specific marker expression through western blot. The results are presented in Fig 6A. Densitometry analysis of the bands was conducted (Fig 6B). AA down regulated CTSK, TRAP and MMP-9 protein expression in differentiating as well as mature osteoclasts (Fig 6B). In contrast, DHA showed a varying degree of inhibition on the studied osteoclastogenic marker expression. While DHA decreased the expression of all the three markers in differentiating osteoclasts, the expression of CTSK and MMP-9 was down-regulated in mature osteoclasts with TRAP expression remaining unaffected (Fig 6B). Undifferentiated monocytes receiving no RANKL expressed high levels of MMP-9 in line with previous observations [36].

### Effects of arachidonic acid and docosahexaenoic acid on osteoclast-specific gene expression

**Fig 7:**
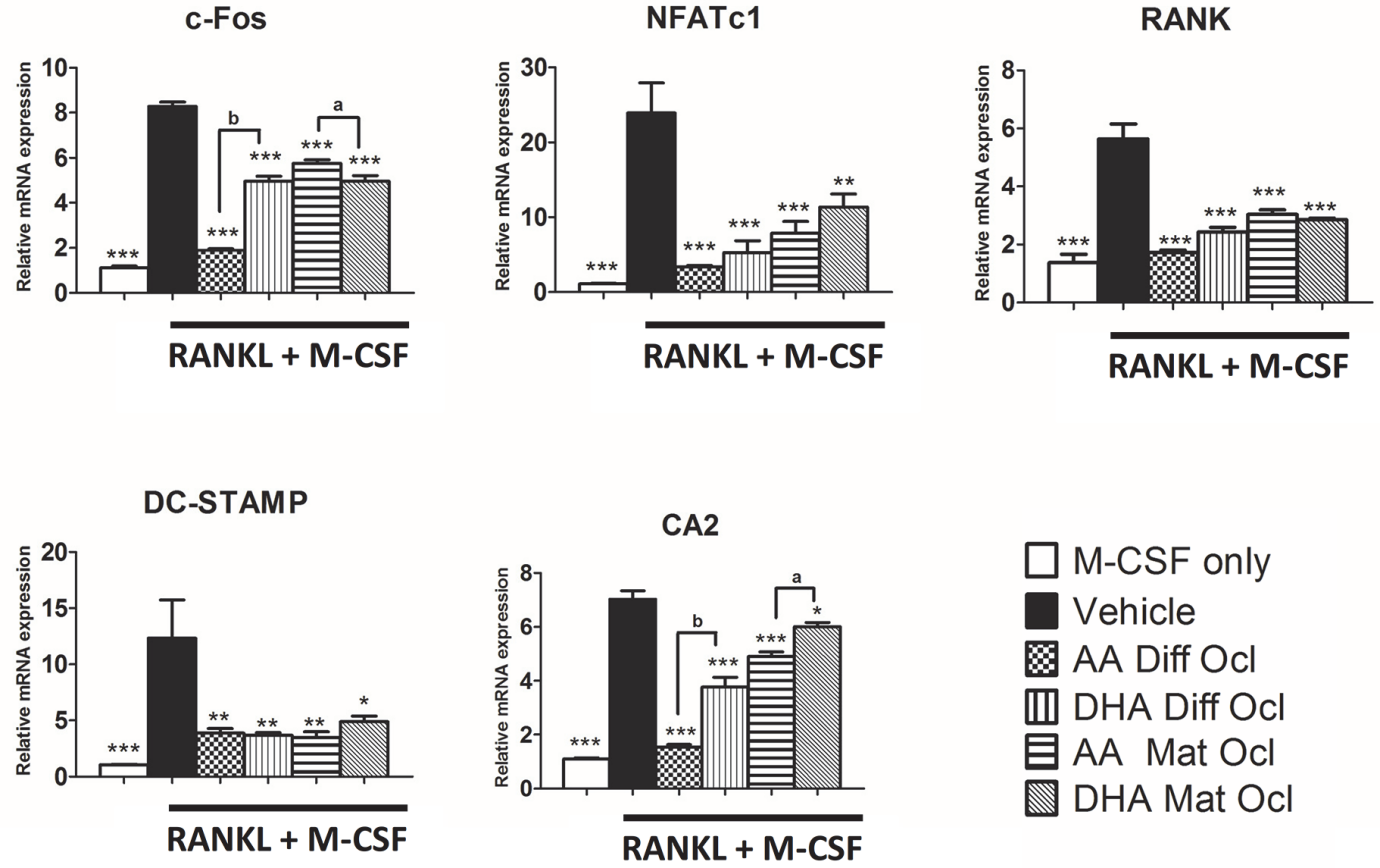
Effects of AA and DHA on osteoclast-specific gene expression. Osteoclast differentiation was stimulated in CD14+ monocytes by the addition of 25 ng ml^-1^ M-CSF and 30 ng ml^-1^ RANKL as described in Materials and Methods. Vehicle (0.08% ethanol) or LCPUFAs (40 μM) were added from day 3 in differentiating osteoclasts and from the onset of resorption (day 12–14) in mature osteoclasts. Experiments were terminated after 3 weeks. Gene expression was quantified by real time-PCR. The results are representative of three independent experiments conducted in triplicate. Diff Ocl—differentiating osteoclasts. Mat Ocl—mature osteoclasts. *P<0.05; **P<0.01; ***P<0.001 vs. vehicle. (a) P<0.05; (b) P<0.001 AA compared with DHA.

Binding of RANKL to its receptor RANK induces expression of downstream signalling molecules that play an indispensable role in osteoclastogenesis. To analyse the effects of the tested LCPUFAs on the expression of various RANKL-induced osteoclast modulators we conducted a qRT-PCR assay. The expression of genes involved in osteoclast activation, (c-Fos, NFATc1) osteoclast formation (RANK, DC-STAMP) and osteoclast activity (CA2) were analysed. The results are presented in Fig 7. All the genes tested showed a significant reduction when exposed to either LCPUFA at 40 μM in differentiating and mature osteoclasts. AA showed a stronger inhibitory effect than DHA on the expression of c-Fos in differentiating osteoclasts and on the expression CA2 in differentiating and mature osteoclasts. DHA showed a greater inhibitory effect on c-Fos expression in mature osteoclasts.

## Discussion

The role of LCPUFAs in bone health has been studied extensively. However, the effects of LCPUFAs on human osteoclast formation and function have yet been reported. In this study the effects of an ω-6 LCPUFA (AA) and an ω-3 LCPUFA (DHA) were examined on human CD14+ derived osteoclast formation, bone resorption and osteoclastogenic marker expression. The present study aimed to gain a better understanding of the effects of these fatty acids on human physiology. To the best of our knowledge, this is the first report to study the effects of LCPUFAs on human CD14+ monocytes in differentiating and mature osteoclasts.

Our findings showed that the tested LCPUFAs can decrease TRAP activity, osteoclast numbers, resorption, and the expression of the genes MMP-9, TRAP, CTSK, c-Fos, NFATc1 and DC-STAMP in differentiating osteoclasts. These findings are consistent with *in vitro* studies on animal cells previously reported [25, 26, 27, 28]. Although both LCPUFAs showed an effect on osteoclastogenesis in differentiating osteoclasts, the effect of AA was more pronounced than that of DHA at equal concentrations. Although smaller and fewer osteoclasts were generated when exposed to LCPUFAs, the resultant osteoclasts showed no other morphological differences compared to the control as they still formed intact actin rings. Interestingly, both LCPUFAs inhibited bone resorption in mature osteoclasts without affecting TRAP+ cell numbers, osteoclast morphology or actin ring formation.

Allard-Chamard *et al*. have shown that AA in combination with a cytosolic phospholipase A_2_ (cPLA_2_) inhibitor (CAY 10502) did not affect actin ring formation or osteoclast viability in mature osteoclasts [37]. This finding corroborates with our results on the effects of AA on actin ring formation. cPLA_2_ is required to liberate the LCPUFAs from the cell membrane [38]. This may indicate that the LCPUFAs while present in the cell membrane may exert their effects in mature osteoclasts possibly by affecting the biophysics of lipid rafts thereby modulating membrane proteins and downstream signalling pathways. Studies have shown that the incorporation of ω-3 LCPUFAs into the cellular membrane is known to affect toll-like receptor 4 (TLR4) which can down-regulate NF-κB expression [38]. Down-regulation of NF-κB may have an effect on osteoclasts as it is known to activate osteoclast formation and function through the activation of NFATc1 [39], the master regulator of osteoclast formation and function.

Our data from differentiating and mature osteoclasts showed that the expression of c-Fos and NFATc1 was decreased by AA and DHA. As c-Fos is upstream of NFATc1, it could be speculated that the decrease in c-Fos might lead to decreased NFATc1 expression. A decrease in c-Fos expression could indicate that the LCPUFAs target upstream proteins such as NF-κB, JNK1 or ERK1 which are known to activate c-Fos during RANKL induced osteoclastogenesis. DC-STAMP, which plays a role in cell-to-cell fusion and is downstream of NFATc1 [10], was also down-regulated by both LCPUFAs in differentiating and mature osteoclasts in this study. As cell-to-cell fusion is required for osteoclast formation, this could likely explain the decrease in the number of osteoclasts formed in differentiating osteoclasts exposed to the LCPUFAs. The RANK gene expression was also found to be down-regulated by both the LCPUFAs in this study. A decrease in the expression of RANK may also in part explain the results observed in this study as this could disrupt the effectiveness of RANKL. Mature osteoclasts exposed to AA showed a greater inhibition of CA2 expression than DHA. CA2 plays a role in acidification of the resorption lacunae. An acidic environment is required for the function of the lysosomal enzymes CTSK and MMP-9, and this could further explain why DHA has a lesser effect on resorption than AA.

CTSK, TRAP and MMP-9 are all proteins that affect resorption [40]. Cathepsins play a role in the breakdown of bone collagen and other organic components of bone [41]. Of the known cathepsins, CTSK is the most abundantly expressed in humans and high levels are found in the resorption lacunae [42]. Human patients with a mutation in the CTSK gene are known to develop pycnodystosis, a condition of abnormally dense bones [43]. CTSK and the degraded products are endocytosed into the osteoclast where they fuse with vesicles containing TRAP [44]. TRAP is then cleaved into an activated form by CTSK and may play a role in cell adhesion and reactive oxygen species generation (both required for resorption) [44, 45]. High levels of MMP-9 are also found in the resorption lacunae [46]. However, MMP-9 deficient mice only show brief disruptions in bone resorption, indicating that CTSK may be more vital for resorption than MMP-9 [47]. Our findings demonstrate that both LCPUFAs can decrease CTSK levels in differentiating and mature osteoclasts when compared to the control. As osteoclastogenesis was decreased in differentiating osteoclasts, lower CTSK levels were expected. However, the lower levels of CTSK could also explain the decrease in resorption by mature osteoclasts exposed to the LCPUFAs. Mature osteoclasts exposed to DHA showed no effect on TRAP expression and expressed higher amounts of MMP-9 than those exposed to AA. The higher concentrations of TRAP and MMP-9 could explain why DHA had a lesser effect on resorption than AA. VNR is required for osteoclasts adhesion to bone as well as osteoclast migration [48]. In this study, both LCPUFAs also decreased the expression of VNR. As both adhesion and migration are needed for resorption, this result may explain the anti-resorptive effects of the LCPUFAs witnessed in our study.

Although, ω-6 LCPUFAs have shown beneficial effects on bone health [21], many studies have suggested that the pro-inflammatory ω-6 LCPUFAs may in fact increase bone loss [49] while the anti-inflammatory ω-3 LCPUFAs improve bone health [19]. However, as the mechanisms of action are not fully understood, the debate still remains controversial. The stimulatory role of inflammatory cytokines on bone resorption is widely accepted in the literature [50, 51]. Prostaglandin (PG) E_2_ is one such cytokine that is a cyclooxygenase (COX) metabolite of AA. However, PGE_2_ has been shown to have biphasic effects on bone with high levels showing negative effects on bone health while low level exposure may have beneficial effects [52]. Lipoxins and resolvins are anti-inflammatory lipid mediators formed by lipoxygenase (LOX) metabolism of ω-6 and ω-3 LCPUFAs respectively [53]. Both are known to inhibit inflammatory bone resorption [53]. Lipoxins have been shown to down-regulate tumor necrosis factor (TNF)-α induced NF-κB activation thereby affecting NFATc1 activation [54]. The differing effects of these mediators may contribute to the conflicting effects of LCPUFAs on bone. In our study, we found that the ω-6 LCPUFA, AA, had a much stronger inhibitory effect on the osteoclasts than the ω-3 LCPUFA, DHA. This may indicate that at the concentrations used in this study, LOX metabolism of the LCPUFAs, particularly in ω-6 LCPUFAs, could have been favoured over COX metabolism. Further studies are needed to elucidate the exact effects of the LCPUFAs in osteoclasts.

This study is the first to show that LCPUFAs can affect osteoclast formation in differentiating osteoclasts as well as the activity of mature human osteoclasts. Therefore, we hypothesize that reduced formation in differentiating osteoclasts and reduced osteoclast activity in mature osteoclasts by modulating osteoclast-specific genes may be a mechanism by which LCPUFAs prevent bone loss. Further studies evaluating the effect of LCPUFAs *in vivo* in mice would be helpful in understanding the complex roles of these fatty acids in human and animal physiology. The addition of COX and LOX inhibitors could also help determine whether the LCPUFAs indeed affect osteoclast activity through their mediators. This knowledge would further establish the molecular actions of LCPUFAs in osteoclasts and confirm putative beneficial effects.

## Supporting Information

S1 Fig Effects of AA and DHA on cell viability. CD14+ monocytes were treated with indicated concentrations of AA and DHA for 48 h and cell viability was measured by alamar blue assay. The results are representative of two independent experiments conducted in triplicate and expressed as percentage cell viability relative to the control.
(TIF)
