# Supplementary figures and images for: Arachidonic Acid and Docosahexaenoic Acid Suppress Osteoclast Formation and Activity in Human CD14+ Monocytes, In vitro
Source: PLoS One. 2015 Apr 13;10(4):e0125145. doi: 10.1371/journal.pone.0125145 (PMC4395026; doi:10.1371/journal.pone.0125145)

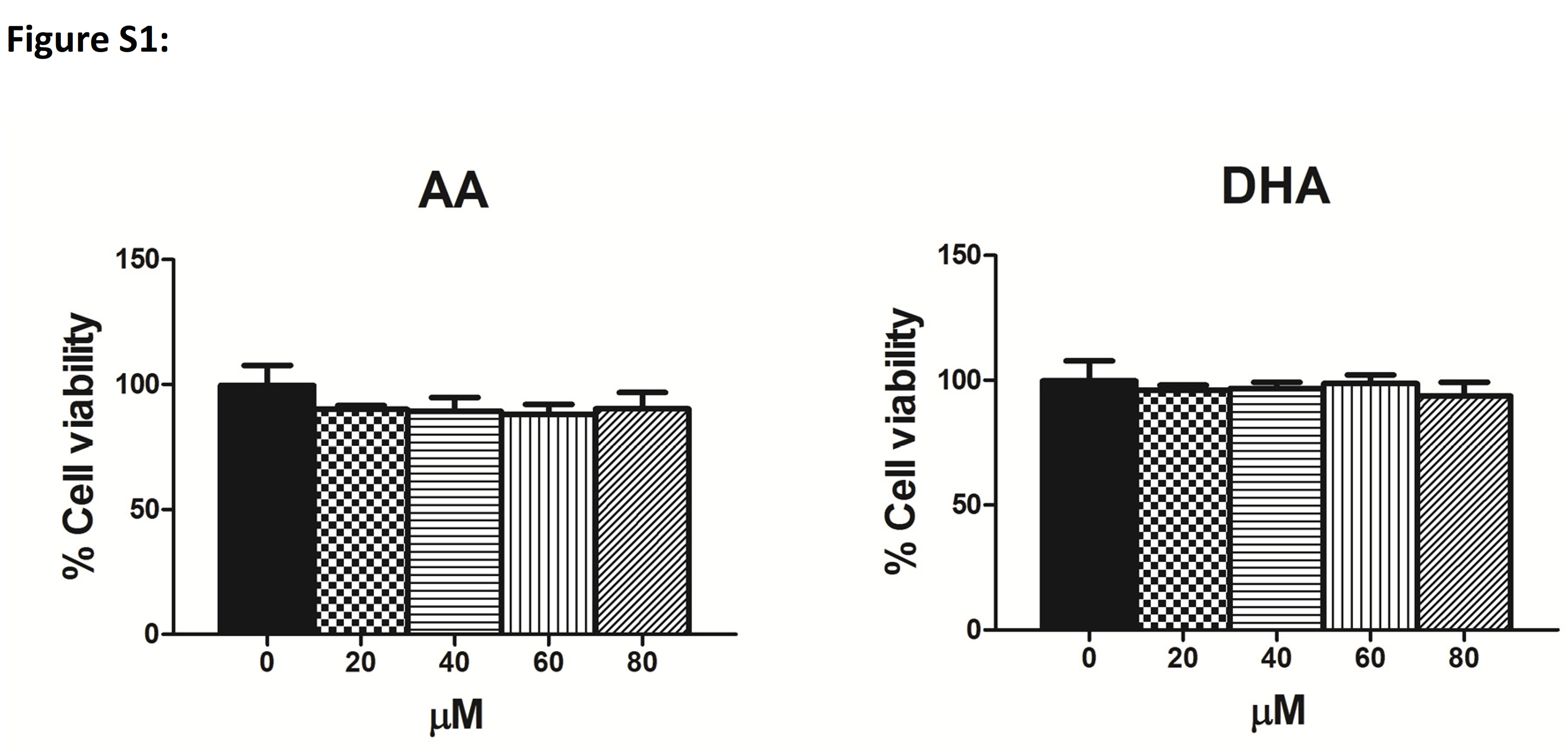

Supplement: S1 Fig — CD14+ monocytes were treated with indicated concentrations of AA and DHA for 48 h and cell viability was measured by alamar blue assay. The results are representative of two independent experiments conducted in triplicate and expressed as percentage cell viability relative to the control. (TIF) [file pone.0125145.s001.tif]
